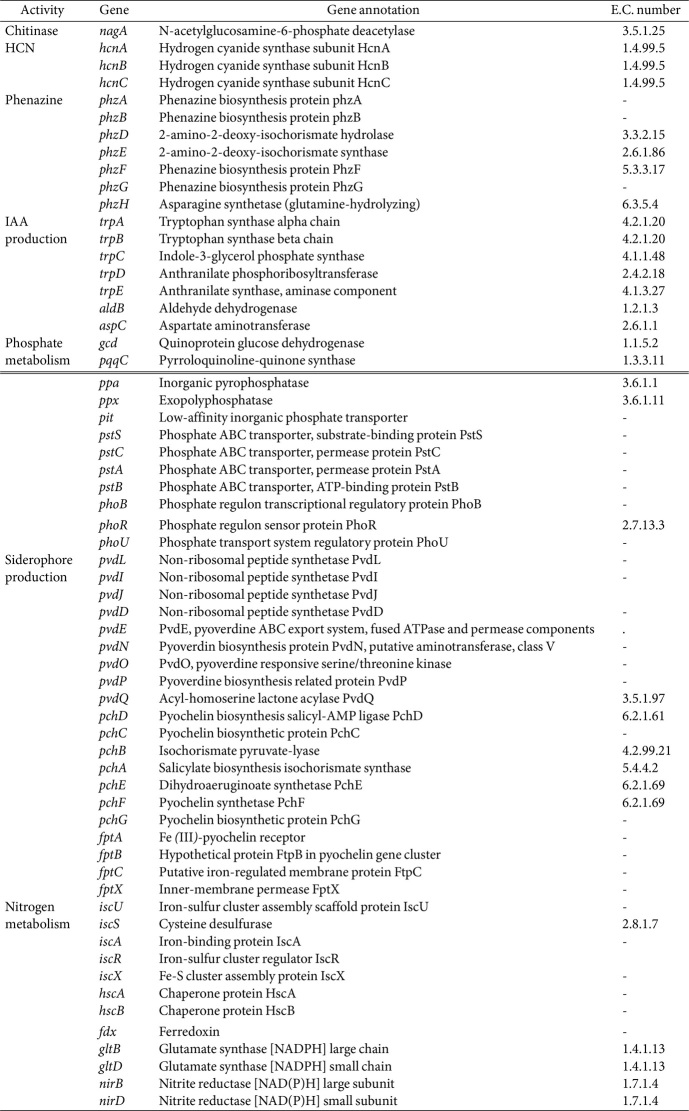# Biocontrol and Growth Promotion of Rice by *Pseudomonas aeruginosa* SNTKU16: Beneficial Properties and Genomic Potential

**DOI:** 10.4014/jmb.2411.11067

**Published:** 2025-02-14

**Authors:** Sutticha Na-Ranong Thammasittirong, Anon Thammasittirong, Sukanya Saechow

**Affiliations:** 1 Department of Science and Bioinnovation, Faculty of Liberal Arts and Science, Kasetsart University, Kamphaeng Saen Campus, Nakhon Pathom 73140, Thailand; 2 Microbial Biotechnology Unit, Faculty of Liberal Arts and Science, Kasetsart University, Kamphaeng Saen Campus, Nakhon Pathom 73140, Thailand

**Keywords:** Biocontrol, plant growth-promoting, genome analysis, rice disease, *Pseudomonas aeruginosa*, seed germination

## Abstract

Dirty panicle and sheath blight are important diseases that have the capacity to reduce rice productivity and grain quality. The bacterial strain SNTKU16 was isolated from soil in a sugarcane field and identified as *Pseudomonas aeruginosa*. This bacterium and its cell-free culture exhibited strong fungal antagonistic activity against a range of rice dirty panicle pathogens (*Curvularia lunata*, *Fusarium semitectum*, and *Helminthosporium oryzae*) and a sheath blight pathogen (*Rhizoctonia solani*). In addition to its role in disease control, the cell suspension and cell-free culture of this strain significantly enhanced rice seed germination and seedling growth. Furthermore, this bacterium exhibited various plant growth-promoting traits such as indole acetic acid, ammonia and siderophore productions, and phosphate solubilization. Genome analysis of SNTKU16 revealed its genetic potential for controlling plant diseases through direct antagonistic activities against pathogens, as well as indirect mechanisms, such as promoting plant growth. These capabilities suggest a multifaceted approach to disease management and plant growth promotion, making this strain an intriguing candidate for further exploration in sustainable agriculture and microbial biotechnology.

## Introduction

Rice (*Oryza sativa* L.) is a crucial economic crop in several countries and serves as a staple food for over one-half of the world's population. Global rice production is impacted by numerous biotic and abiotic factors. Among these, fungal pathogen infections are a major cause of yield loss. Of the various fungal diseases, dirty panicle is considered a major constraint on rice production [1]. Dirty panicle disease is caused by a complex infection involving multiple phytopathogenic fungi such as *Alternaria padwickii*, *Curvularia lunata*, *Fusarium verticillioides*, *Fusarium semitectum*, and *Helminthosporium oryzae* [2, 3]. The infection begins at the early boot stage and continues through the harvest period. Infected rice seeds serve as a source of inoculum for these pathogenic fungi, facilitating their spread from seed storage to new crops. This disease has substantial impacts on rice production, causing both qualitative and quantitative yield reductions [2]. Sheath blight, caused by *Rhizoctonia solani*, is also a serious disease affecting rice in major rice-growing regions. *R. solani* infects and destroys leaf sheath and leaf blades and can attack the plant at any growth stage. This necrotrophic fungus reduces rice productivity and grain quality. Additionally, it is a severe pathogen for other important crops [4]. The application of fungicides is a commonly used strategy for managing rice diseases. However, these chemical agents can induce fungicide resistance in pathogens. Increasing concerns about pesticide residues on rice and their potential risks to human health and the environment are driving the search for safer and more environmental-friendly crop protection methods [1].

*Pseudomonas* is one of the most abundant bacterial genera in soil ecosystems, known for its diverse ecological roles. The species *Pseudomonas aeruginosa* is a widespread fluorescent pseudomonad in soil. While some strains are opportunistic pathogens, others act as biocontrol agents [5]. *P. aeruginosa* showed antagonistic activity against several plant pathogens, such as *Sporisorium scitamineum*, *Ceratocystis paradoxa* and *Fusarium verticillioides* in sugarcane [6], *Magnaporthe grisea* and *R. solani* in rice, *Colletotrichum capsici* in chilli [7], and *Fusarium oxysporum* and *Ralstonia solanacearum* in tomato [8]. Furthermore, *P. aeruginosa* has been shown to promote tomato and Indian mustard seedling growth [9, 10]. This species produces a range of antimicrobial compounds, including phenazine, pyrrolnitrin, hydrogen cyanide (HCN), and hydrolytic enzymes [6, 8, 11, 12]. In addition, it synthesizes plant growth-promoting substances such as indole acetic acid (IAA), siderophore and ammonia [8, 10, 11].

In the current study, *P. aeruginosa* SNTKU16 was isolated from sugarcane field soil and selected based on its strong antifungal activity. The effects of this antagonistic bacterium and its cell-free culture on the biocontrol of a broad range of pathogenic fungi responsible for dirty panicle and sheath blight diseases were evaluated. Additionally, its production of hydrolytic enzymes and plant growth-promoting traits were investigated. To our knowledge, this is the first report demonstrating that both *P. aeruginosa* SNTKU16 cells and its cell-free culture can function as biocontrol agents against rice pathogens, as well as promote rice seed germination and seedling growth. Furthermore, genome analysis provides a foundation for future studies to understand the mechanisms by which this bacterium functions as a biocontrol agent and plant growth promoter.

## Materials and Methods

### Bacterial Isolation

Soil bacteria were isolated from rice paddy fields and sugarcane fields in Nakhon Pathom and Suphanburi provinces, Thailand. To enrich the bacterial cultures, 10 g of soil was added to 100 ml of nutrient broth (NB) and incubated at 37°C for 48 h with shaking at 150 rpm. After serial dilution, the bacterial suspensions were plated on nutrient agar (NA) and incubated at 37°C for 48 h. Then, the individual bacterial colonies were purified by repeated streaking on fresh NA plates to obtain pure isolates.

### *In Vitro* Screening of Antagonistic Bacteria

The dual culture method, as described by Saechow *et al*. [13] was used to evaluate the inhibition of mycelial growth in several rice pathogenic fungi (*C. lunata* PFR12, *F. semitectum* PFR5, *H. oryzae* PFR3, and *R. solani* KPK00289) by isolated bacteria. These pathogenic fungi were obtained from the Plant Health Clinic, Kasetsart University, Kamphaeng Saen Campus, Nakhon Pathom, Thailand. The growth of the pathogens on potato dextrose agar was measured and compared to the controls, which were inoculated only with the fungal pathogen. The percentage of inhibition of radial growth (PIRG) was calculated using the formula:

PIRG (%) = [(R1 – R2) / R1] × 100

where, R1 is the radial diameter of the control colony and R2 is the radial diameter of the treatment colony. Experiments were performed in triplicate.

### Bacterial Cell-Free Culture Preparation

SNTKU16 was grown overnight in NB at 37°C with shaking at 150 rpm. The culture was adjusted to an optical density at 600 nm (OD_600_) of 0.5 and then 1% of this adjusted culture was inoculated into fresh NB and shaken at 150 rpm for 72 h at 37°C. Next, bacterial cells were collected using centrifugation at 10,000 ×*g* for 10 min at 4°C. The supernatant was then passed through a 0.22 μm Millipore filter to obtain a cell-free culture.

### Effect of SNTKU16 Cell-Free Culture on Antifungal Activity

Fungal growth inhibition assay was performed using the dry weight determination method according to Saechow *et al*. [13]. The cell-free culture was added to 100 ml of potato dextrose broth (PDB) containing a spore suspension of fungal pathogen at a concentration 1 × 10^6^ spores/ml. This mixture was prepared to yield final concentrations of 5%, 10%, 15%, 20%, 30% and 50% cell-free culture. PDB medium without cell-free culture served as the control. The cultures were incubated at 30°C with shaking at 150 rpm for 7 days. The mycelia were collected using filtration and dried at 55°C for 5–7 days until a constant weight was achieved.

### Effect of SNTKU16 on Rice Seed Germination and Seedling Growth

The effects of the cell suspension and cell-free culture of SNTKU16 on rice seed germination and seedling growth were evaluated according to Sripodok *et al*. [14], with slight modifications. Rice seeds (cultivar Suphanburi 1) were soaked in distilled water for 15 h and surface-sterilized by submerging for 10 min in 10%Clorox. After washing five times with sterilized distilled water, the sterilized seeds were immersed for 1 h in 50%cell suspension and 50% cell-free culture of SNTKU16. Seeds treated with sterilized distilled water and NB broth medium were used as the control. All the seeds were blotted dry and then placed on moist filter paper blotters and incubated in a growth chamber. Seed germination and length of shoot and root were measured every 24 h for 7 days. A seed vigor index was calculated using the formula [15].

Seed vigor index = %Germination × (shoot length + root length)

### Extracellular Hydrolytic Enzyme Productions

The qualitative assay for extracellular hydrolytic enzyme production was conducted following Sripodok *et al*.[14], with slight modifications. The ability of SNTKU16 to produce amylase, lipase, protease and cellulase was assessed using starch, Tween 80, skimmed milk and carboxymethyl cellulose (CMC) agar plates, respectively, via point inoculation followed by incubation at 37°C for 48 h. Chitinase activity was evaluated on colloidal chitin agar and incubated at 37°C for 120 h. Enzyme activity was visualized by flooding the starch agar and CMC agar plates with a 1.5% iodine solution for 5 min. Positive results were indicated by a clear halo zone around the colony. For chitinase and protease, a clear halo zone without staining signified positive results. A precipitation zone around the colony indicated lipase activity.

### IAA Production

SNTKU16 was cultured in NB broth supplemented with 500 μg/ml of L-tryptophan for 48 h at 37°C with shaking at 150 rpm. A cell-free culture was quantified for IAA using Salkowski reagent according to Saechow *et al*.[13]. Optical density of the developing pink color was measured spectrophotometrically at 530 nm. A series of pure IAA (Sigma-Aldrich Co., USA) was established as a standard curve.

### Phosphate Solubilization

SNTKU16 was inoculated on National Botanical Research Institute’s phosphate growth medium (NBRIP) agar containing 0.5% Ca_3_(PO_4_)_2_ using point inoculation followed by incubation at 37°C for 14 days. A halo zone around the bacterial colony indicated phosphate solubilization [14]. For the quantitative assay, SNTKU16 was inoculated into NBRIP broth and incubated at 37°C for 14 days with shaking at 150 rpm. The cell-free culture was quantified for solubilized phosphate using the ascorbic acid method [13, 16].

### Siderophore Production

Siderophore production was investigated using chrome azurol S (CAS) with modified Gaus No. 1 medium (MGs) agar [17]. Colonies producing yellow, orange, or red zones were considered positive for siderophore production. Siderophore production was quantitatively evaluated using the method described by Arora and Verma [18], with some modifications. SNTKU16 was inoculated into MGs broth and incubated at 37°C with shaking at 150 rpm for 48 h. A 0.5 ml sample of the culture supernatant was mixed with 0.5 ml of CAS reagent; after 20 min, the absorbance was measured at 630 nm.

Siderophore production was quantified as the percentage of siderophore units (psu), calculated using the formula:

Siderophore units (psu)= [(Ar – As) / Ar] × 100

where Ar is the absorbance of the reference (CAS solution and uninoculated broth) and As is the absorbance of the sample (CAS solution and cell-free supernatant).

### Ammonia Production

SNTKU16 was cultured in peptone water broth and incubated at 37°C for 48–72 h. Ammonia production was indicated by the development of a yellow-to-brown color after adding Nessler’s reagent [19].

### Genome Sequencing and Analysis

Genomic DNA was extracted using a BioFact^TM^ Genomic DNA Prep Kit (BioFact, Republic of Korea) according to the manufacturer’s instruction. DNA quality was evaluated using agarose gel electrophoresis and the DNA quantity and purity were measured using a DS-11 FX+ spectrophotometer (DeNovix Inc., USA). The SNTKU16 draft genome was sequenced at Celemics Inc., (Republic of Korea) using the Illumina NextSeq 550 platform (Illumina, USA).

De novo sequence assembly of the genome was performed using the Unicycler software [20]. The tRNA, rRNA, tmRNA, GC content, and protein-coding sequences (CDSs) were predicted using the Prokka gene annotation tool [21]. The draft genome of SNTKU16 was analyzed for gene prediction using the Rapid Annotation using Subsystem Technology (RAST) server [22]. The Clusters of Orthologous Groups (COGs) were assigned using eggNOG-mapper v2 [23]. The circular map of the SNTKU16 draft genome was created using Proksee software [24].

### Comparative Genomics and Phylogenomics

The average nucleotide identity (ANI) based on BLASTN (ANIb) between genomes was calculated using the JspeciesWS software [25], applying the nucleotide BLAST algorithm with default parameter settings. Digital DNA-DNA hybridization (dDDH) values were determined using a genome-to-genome distance (GGDC) server [26]. Phylogenetic analysis of the complete genome was conducted using the Bacterial Genome Tree Service within the Bacterial Bioinformatics Resource Center (BV-BRC) platform, applying the codon tree method [27].

### Statistical Analysis

Data were analyzed using analysis of variance (ANOVA) with SPSS 16.0 software (SPSS Inc., USA). Duncan's multiple range post hoc test was used for pairwise comparisons to identify significant differences. Results are presented as mean ± standard deviation, with statistical significance set at *p* < 0.05.

## Results

### Effect of SNTKU16 and Its Cell-Free Culture on Antifungal Activity

**Fig. 1:**
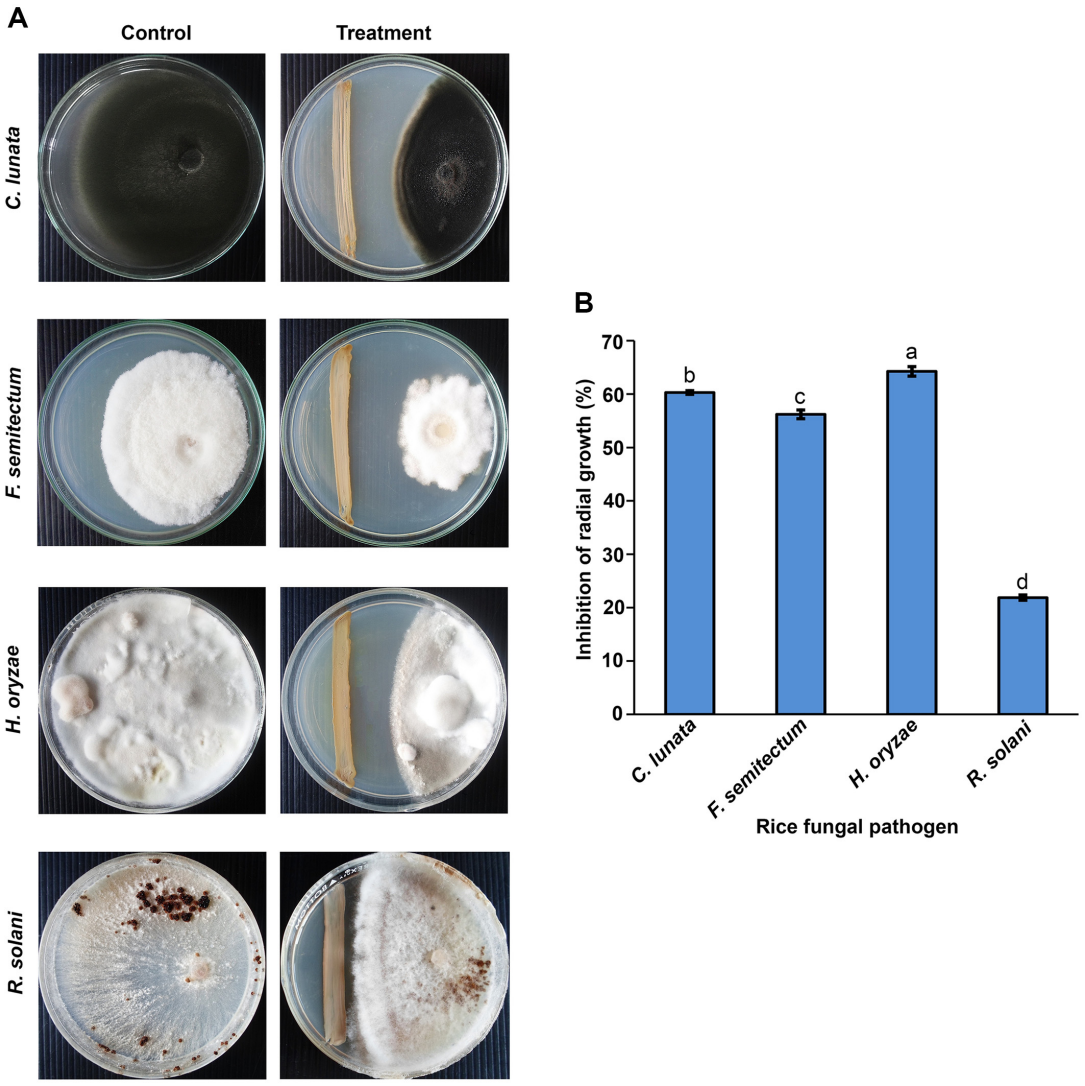
Antagonistic activity of SNTKU16 on mycelial growth inhibition of rice pathogens using dual culture method (A) and percentage of radial growth inhibition (B). Values represent mean ± standard deviation. Means with different lowercase superscript letters indicate significant differences among treatments at *p* < 0.05.

**Fig. 2:**
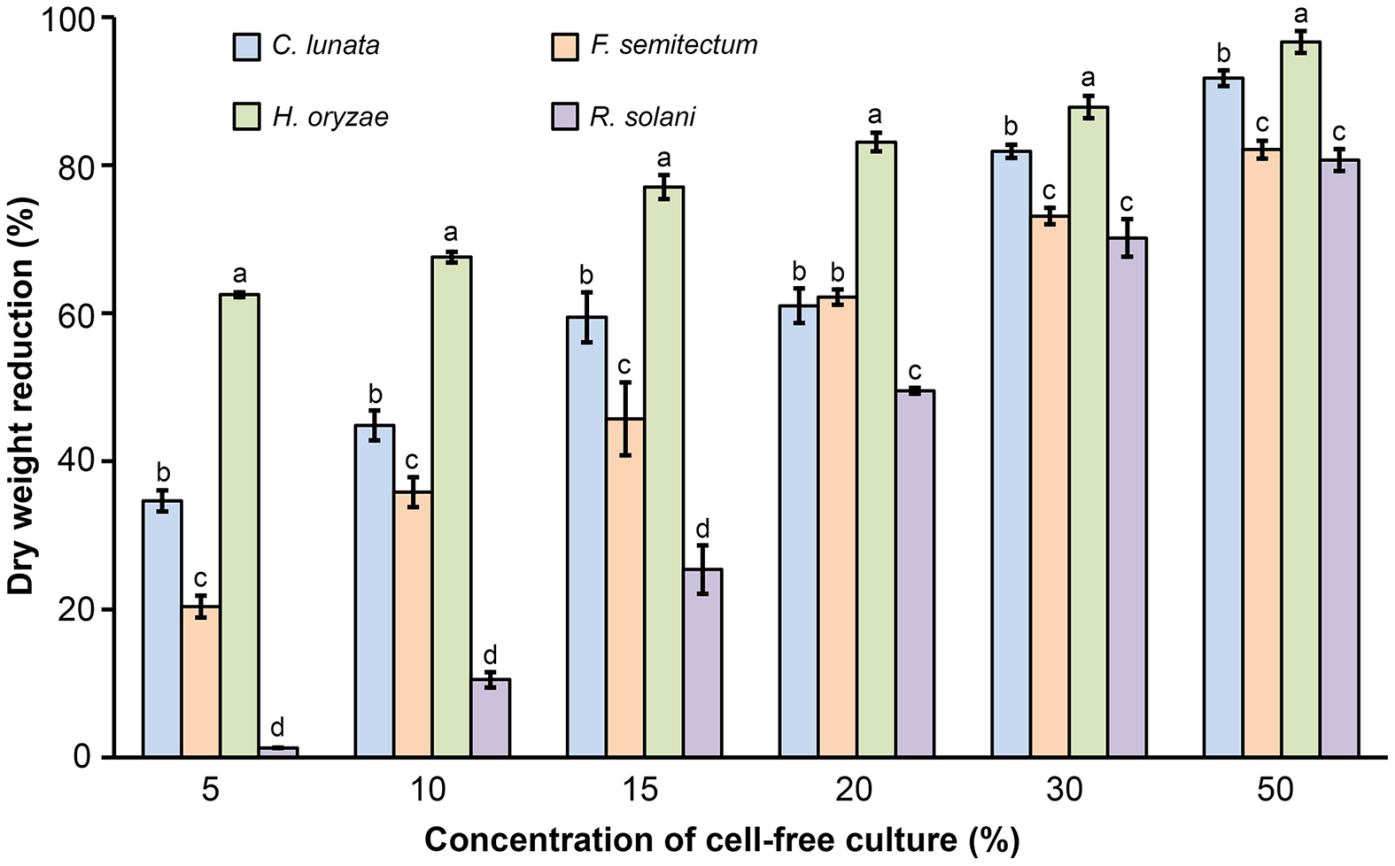
Antagonistic activity of SNTKU16 cell-free culture on mycelial growth inhibition of rice pathogens using dry weight determination method. Values represent mean ± standard deviation. Means with different lowercase superscript letters indicate significant differences in each concentration of cell-free culture at *p* < 0.05.

Among 53 bacterial isolates, SNTKU16, obtained from sugarcane field soil, was selected for its strong antagonistic activity against a wide range of rice pathogenic fungi. SNTKU16 effectively inhibited the mycelial growth of rice pathogens responsible for dirty panicle disease, including *C. lunata*, *F. semitectum*, and *H. oryzae*, as well as the sheath blight pathogen, *R. solani* (Fig. 1). The inhibitory effect of the cell-free culture of SNTKU16 on fungal growth was assessed using the dry weight determination method. Based on the results, the cell-free culture of SNTKU16 considerably significantly inhibited the growth of rice pathogenic fungi (Fig. 2). The degree of inhibition was directly proportional to the concentration of the cell-free culture. Among the pathogens tested, the cell-free culture of SNTKU16 exhibited the most pronounced inhibitory effect on *H. oryzae*, which was similar to the results observed in the dual culture assay. The highest inhibition rate of *H. oryzae* was 96.64% when treated with a 50% concentration of cell-free culture.

### Evaluation of Production of Hydrolytic Enzymes and Plant Growth-Promoting Traits of SNTKU16

**Fig. 3:**
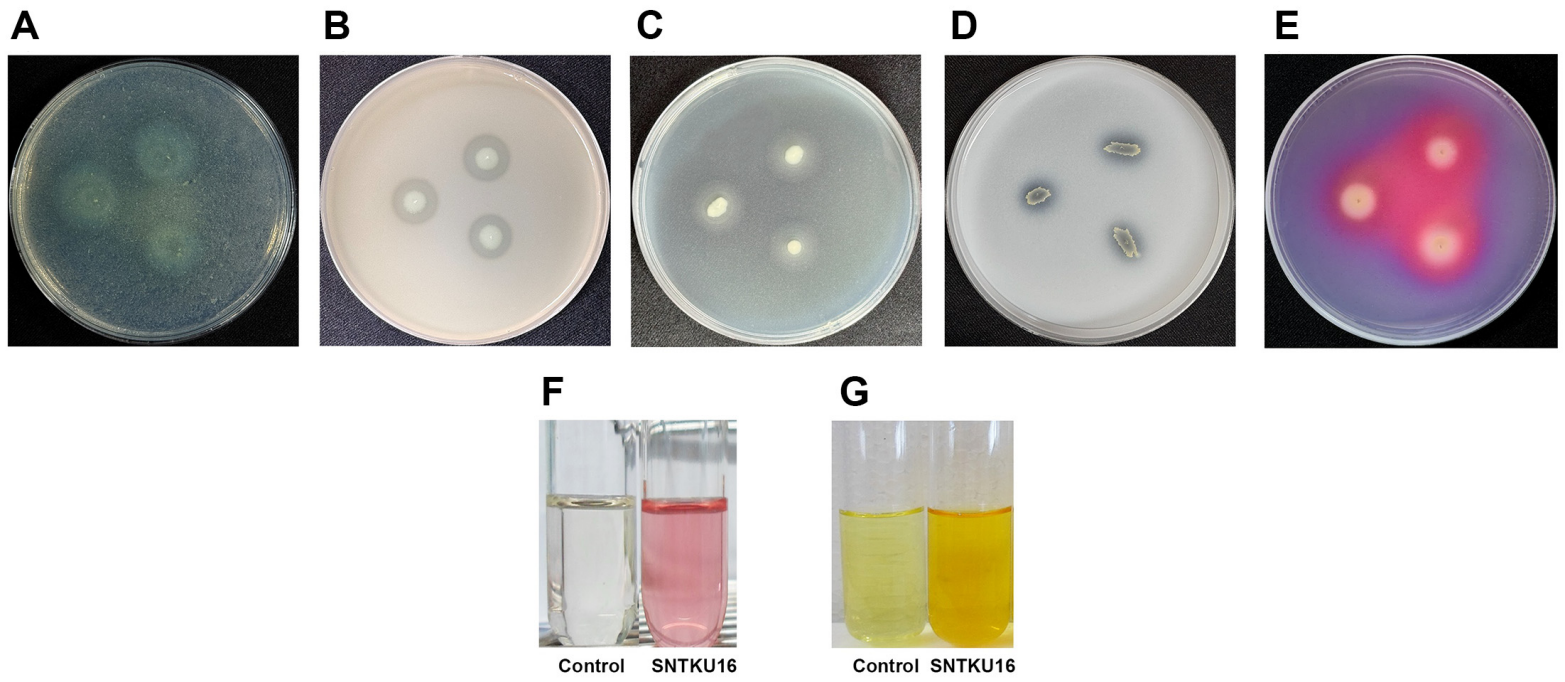
*In vitro* assessment of hydrolytic enzyme activities and plant growth-promoting traits of SNTKU16: chitinase activity (A) protease activity (B) lipase activity (C) phosphate solubilization (D) siderophore production (E) IAA production (F) and ammonia production (G).

Extracellular enzymatic lysis is one of the mechanisms for inhibiting pathogenic microorganisms [28]. Based on the current results on the production of extracellular enzymes, SNTKU16 produced chitinase, protease, and lipase (Table 1 and Fig. 3A-3C). Clear hydrolytic zones on the chitin agar and skimmed milk agar confirmed the activities of chitinase and protease, respectively. The formation of a precipitate surrounding the colony on the Tween 80 agar indicated a positive reaction for lipase enzyme detection.

SNTKU16 was also showed for its plant growth-promoting traits, as shown in Table 1 and Fig. 3D-3G. SNTKU16 exhibited the ability to solubilize tri-calcium phosphate in the NBRIP agar, forming a clear halo around the colony. The maximum phosphate solubilization was 0.58 mg/l in the NBRIP liquid medium. SNTKU16 demonstrated siderophore production, indicated by a change from a bluish to a red-orange color and the formation of a halo zone on chrome azurol S agar. Quantitative analysis revealed that strain SNTKU16 produced siderophore at 37.70 psu. Additionally, SNTKU16 synthesized IAA at 28.82 μg/ml. Ammonia production was confirmed by the development of a yellow-to-brown color in peptone water broth.

### Effect of SNTKU16 and Its Cell-Free Culture on Rice Seed Germination and Seedling Growth

**Fig. 4:**
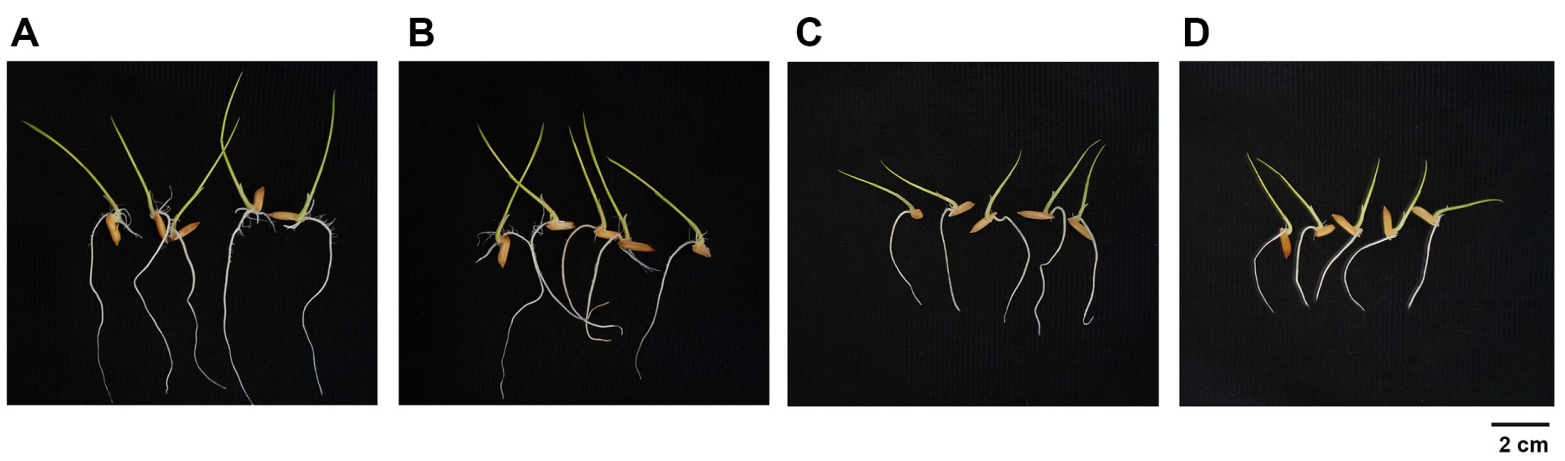
Rice seedlings growth at 7 days after treatment with 50% SNTKU16 cell suspension (A) 50% SNTKU16 cell-free culture (B) NB medium (C) and water (D).

The impact of cell suspension and cell-free culture of SNTKU16 on rice seed germination and seedling growth was examined. Compared to the control, there were significant improvements in both germination and growth when treated with cell suspension and cell-free culture of SNTKU16 (Table 2 and Fig. 4). The maximum values for the germination rate of seed, shoot length, root length and seed vigor index of the seed treated with cell suspension and cell-free culture of SNTKU16 were 98.22–98.90%, 3.95–4.27 cm, 5.33–6.79 cm and 911.42–1,093.92%, respectively, whereas the control group values were 96.66–97.54%, 2.88–2.92 cm, 3.10–4.02 cm, and 578.10–676.98%, respectively.

### General Features of the Genome of SNTKU16

**Fig. 5:**
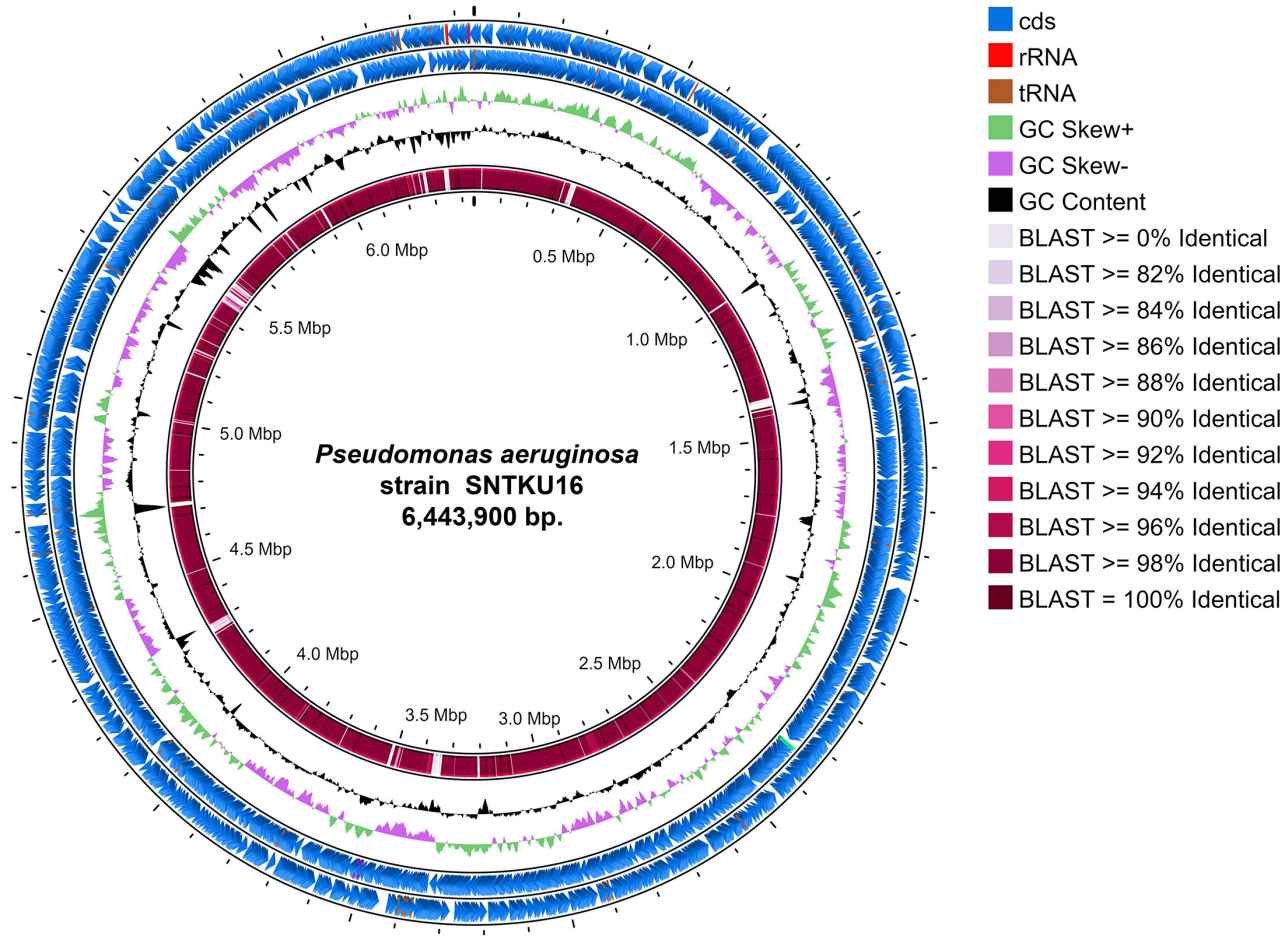
Circular genome map of SNTKU16 visualized and compared with *P. aeruginosa* DSM22644 using Proksee software. From outer to inner circle: protein-coding genes on reverse and forward strand (blue); RNA genes (rRNA, red; tRNA, brown); GC skew (positive, green; negative, purple); GC content (black); and a comparative analysis of SNTKU16 genome against *P. aeruginosa* DSM22644 genome using the BLAST2.12.0 software (pale pink to dark red).

**Fig. 6:**
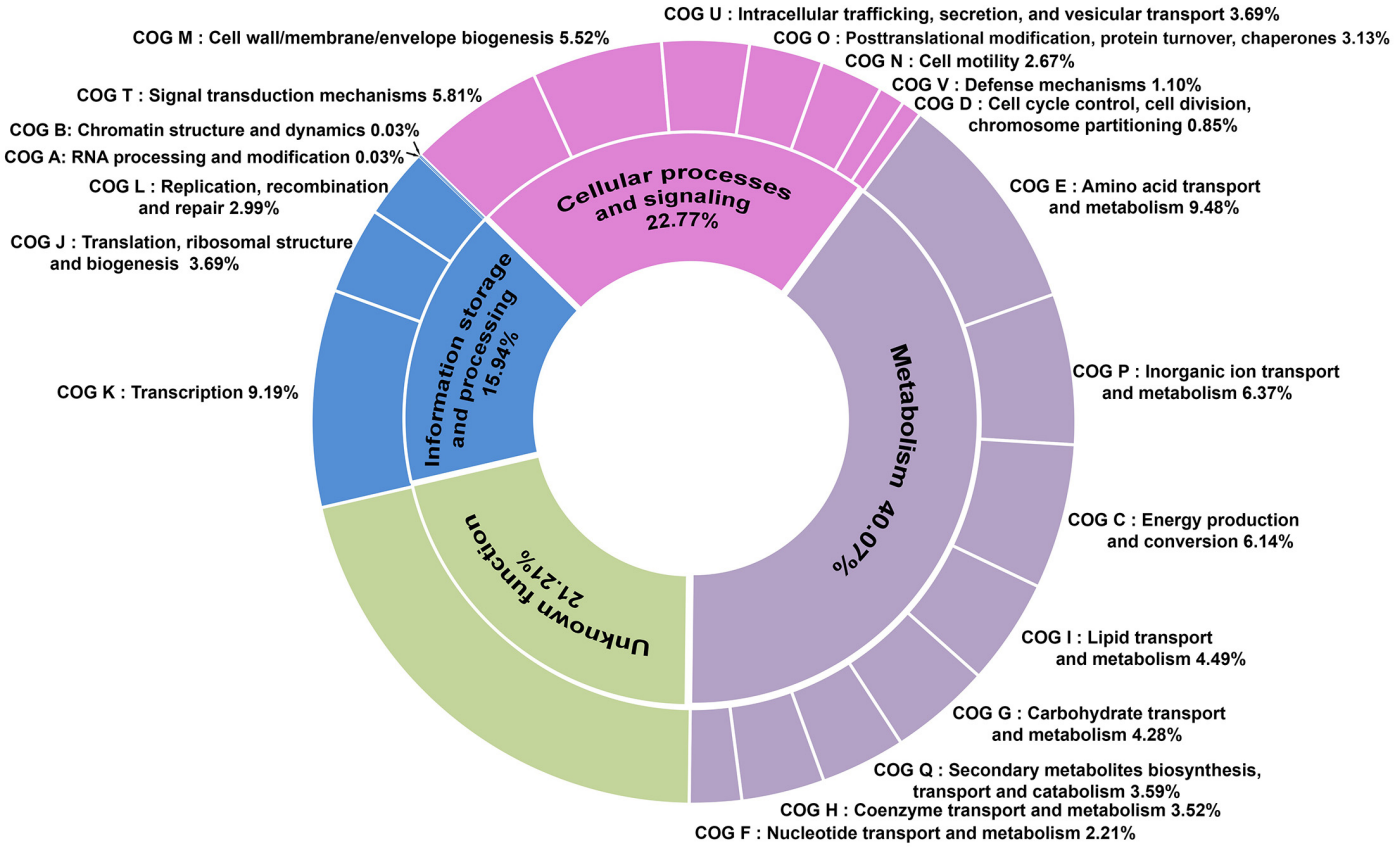
COG functional classification analysis of SNTKU16 genome using eggNOG-mapper.

The draft genome of SNTKU16 is 6,443,900 base pairs (bp) in length, with an average GC content of 66.3%(Fig. 5). The general characteristics were annotated using the Prokka tool as shown in Table 3. A total of 5,966 genes were annotated in the genome, including 5,899 CDSs. The COG database was used to functionally categorize the predicted proteins of the SNTKU16. Out of all the annotated sequences, 5,321 CDSs were assigned to 20 COG categories, providing insights into the organism’s functional potential and metabolic capabilities. The main COG categories were amino acid transport and metabolism (9.48%) and transcription (9.19%), as shown in Fig. 6. The draft genome sequence of SNTKU16 was deposited in the NCBI/GenBank database under the accession number JBEGDM000000000.

### Comparative Genomics and Phylogenomics

The average nucleotide identity (ANI) is a widely used metric for assessing genomic similarity and evolutionary relationships between prokaryotic strains, where an ANI value above 95% generally indicates that the genomes belong to the same species [29]. The ANI values between SNTKU16 and other *P. aeruginosa* strains were in the range 98.38–98.69%, with corresponding dDDH values of 89.40–90.60% (Table 4). These consistent ANI and dDDH values indicated that they belonged to the same species.

**Fig. 7:**
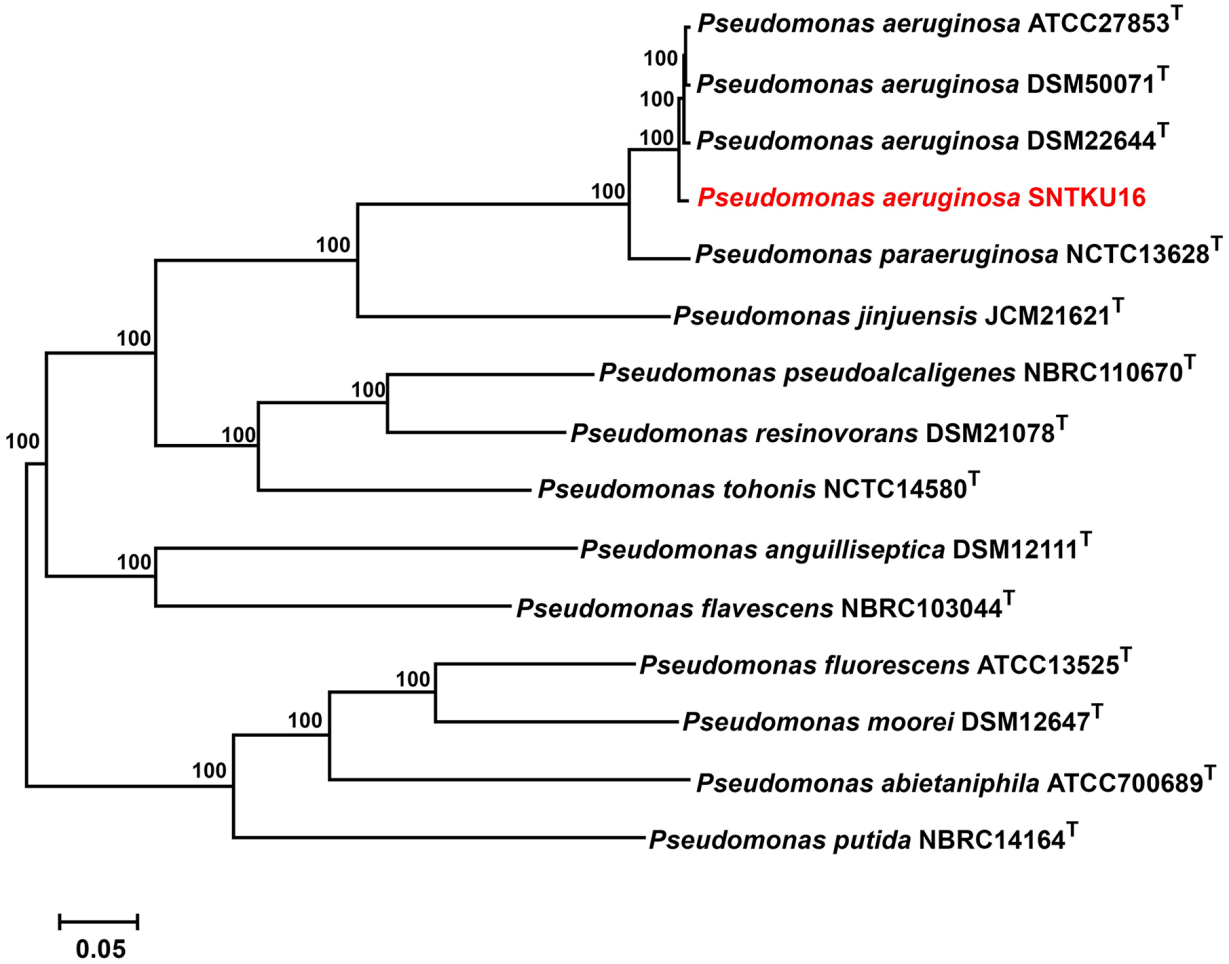
Phylogenomic tree showing SNTKU16 and related type strains of genus *Pseudomonas*.

In addition, phylogenomics demonstrated that SNTKU16 was closely related to *P. aeruginosa* strains (Fig. 7). Comparative genomics and phylogenomics confirmed that SNTKU16 was a member of *P. aeruginosa*, with the closest match being *P. aeruginosa* DSM22644.

### Biocontrol and Plant Growth-Promoting-Related Genes in SNTKU16 Genome

Genome annotation revealed that SNTKU16 harbored a diverse array of genes associated with biocontrol and plant growth-promoting activities (Table 5). As a potential biocontrol strain, genes related to chitinase activity (*nagA*), HCN production (*hcnABC*), and phenazine production (*phzABDEFGH*) were identified. In addition, genes related to tryptophan biosynthesis (*trpABCDE*) and IAA biosynthesis via the indole-3-pyruvate pathway (*aldB* and *aspC*) were identified, suggesting potential roles in auxin production and plant growth promotion. The SNTKU16 genome harbored genes related to phosphate metabolism, including genes for inorganic phosphate solubilization and organic phosphate mineralization (*gcd*, *pqqC*, *ppa*, and *ppx*), phosphate uptake and transport (*pit* and *pstSCAB*), and phosphate starvation regulation (*phoBR*, and *phoU*). The genome also contained genes responsible for siderophore production, such as pyoverdine (*pvdL*, *pvdI*, *pvdJ*, *pvdD*, *pvdE* *pvdN*, *pvdO*, *pvdP*, and *pvdQ*), and pyochelin (*pchDCBA*, *pchEFG*, and *fptABCX*). Furthermore, the genome contained several nitrogen-related genes involved in nitrogen fixation (*iscU*, *iscS*, *iscA*, *iscR*, *hscB*, *hscA*, *fdx*, and *iscX*), ammonia assimilation (*gltBD*) and nitrosative stress response (*nirBD*).

## Discussion

Rice production is severely impacted by fungal diseases, which can greatly reduce yields. The use of beneficial antagonistic bacteria offers a promising alternative to chemical fungicides. In this study, the bacterium SNTKU16 demonstrated significant *in vitro* inhibitory activity against several fungal pathogens responsible for rice diseases. Using the dual culture method, SNTKU16 effectively inhibited mycelial growth of rice fungal pathogens responsible for dirty panicle and sheath blight of rice diseases. Additionally, the antifungal activity of the SNTKU16 cell-free culture effectively inhibited the growth of all the tested rice pathogenic fungi, with its inhibitory effect against pathogens showing a similar trend to the results observed in the dual culture assay. The production of lytic enzymes is a common trait among antagonistic microorganisms. Several *Pseudomonas* species act as fungal antagonists by producing hydrolytic enzymes such as chitinase, cellulase, protease, lipase, and β-glucanase [6, 30]. These enzymes play crucial roles in inhibiting or modifying cell-wall synthesis or degrading the cell walls of host or plant pathogens [31]. *Pseudomonas fluorescens* Pf-5 exhibited a significant positive correlation between the percentage of growth inhibition of *F. oxysporum* f. sp. *cumini* and the activity levels of chitinase, β-1,3-glucanase, and protease enzymes in the culture medium [30]. In the current study, SNTKU16 was positive for chitinase, protease, and lipase activities indicating its potential role in biocontrol mechanisms.

The potential of SNTKU16 to promote plant growth was evaluated using both cell suspension and cell-free culture treatments. Rice seeds treated with these preparations showed significant improvements in seed germination, as well as in shoot and root elongation. These improvements were further quantified by significant increases in the vigor index, a key parameter in evaluating seed quality and its direct correlation to crop productivity. In treated rice seeds, the vigor index was in the range 911.42–1,093.92%, being markedly higher than the values observed in the untreated controls (578.10–676.98%). The use of cell suspension treatment versus cell-free culture treatment presents distinct benefits and challenges. Cell suspensions may offer sustained plant-microbe interactions and colonization; however, they have a shorter shelf life and may require special storage conditions to maintain cell viability. On the other hand, cell-free cultures are safer and easier to use and store, though they may require more frequent applications to maintain their efficacy [32-34]. Overall, this study highlighted the potential of both cell suspension and cell-free culture of SNTKU16 to enhance key plant growth parameters, offering promising solutions for agricultural productivity and sustainability.

Several studies have reported the biocontrol and plant growth promotion abilities of *P. aeruginosa* in agriculture. For example, *P. aeruginosa* has been shown to act as a biocontrol agent in rice against bacterial leaf blight (*Xanthomonas oryzae* pv. *oryzae*) [35], as well as against *Bipolaris oryzae*, *Colletotrichum gloeosporioides*, *Fusarium fujikuroi*, *F. oxysporum*, *Fusarium solani*, and *R. solani* [36]. Ghadamgahi *et al*. [9] demonstrated that *P. aeruginosa* FG106 and its cell-free culture exhibited biocontrol activity against pathogens of potato, tomato and taro, while its cell suspension enhanced tomato seedling growth. Saikia *et al*. [37] found that rice seeds treated with cell suspensions of *P. aeruginosa* strains *Pa*RsG18, *Pa*RsG27, and *Pa*RsG28 not only promoted plant growth but also induced systemic resistance in rice against *R. solani*. Roychowdhury *et al*. [10] reported that the cell suspension of the *P. aeruginosa* strain PGP significantly enhanced seedling growth in *Brassica juncea*. To our knowledge, this is the first study to report both the biocontrol efficacy and plant growth-promoting activities of *P. aeruginosa* in both cellular and cell-free culture forms. This work highlighted that *P. aeruginosa*, through both its viable cells and cell-free supernatant, exhibited broad-spectrum antagonistic activity against rice pathogens, particularly those responsible for dirty panicle and sheath blight diseases. Furthermore, these treatments significantly enhanced rice seed germination and seedling growth, suggesting potential applications in sustainable agriculture.

Based on the biocontrol and plant growth-promoting abilities of SNTKU16, its draft genome was sequenced to explore the genetic basis of these functions. The SNTKU16 genome was 6,443,900 bp with a GC content of 66.3%. It consisted of 5,966 genes, of which 5,899 were protein-coding. Comparative genomic and phylogenomic analyses supported that SNTKU16 was *P. aeruginosa*. Genome analysis revealed that SNTKU16 contained genes associated with various antimicrobial activities and plant growth promotions. Secondary metabolites produced by *Pseudomonas* species, including HCN and phenazine, are recognized for their antagonistic properties. For example, HCN produced by *P. corrugata* CFBP5454 and *P. mediterranea* CFBP5447 demonstrated antifungal activity against *Botrytis cinerea* [38]. HCN synthase is a membrane-bound enzyme that oxidizes glycine, producing HCN and carbon dioxide [39]. Genes encoding HCN synthase (*hcnABC*) were identified in *P. aeruginosa* NG61 [40], *P. aeruginosa* DJ06 [41], and *P. corrugata* CFBP5454 [38], all of which demonstrated HCN production. The current study identified the *hcnABC* gene in the *P. aeruginosa* SNTKU16 genome, suggesting that this strain may also have the ability to produce HCN.

Phenazines are redox-active molecules with heterocyclic ring structures that exhibit broad-spectrum antibiotic properties. Pseudomonad phenazine-producing strains, such as *P. chlororaphis*, *P. synxantha*, *P. yamanorum*, and *P. orientalis*, have been shown to inhibit the growth of various potato pathogens [42]. Phenazine-producing *P. aeruginosa* PUPa3 exhibited broad-spectrum antifungal activity against rice pathogens, including *R. solani*, *Magnaporthe grisea*, and *Sarocladium oryzae* [7]. The genome of the phenazine-producing bacterium *P. chlororaphis* strain HT66 contained the phenazine biosynthesis operon (*phzABCDEFGH*) [43]. The operon *phzABDEFGH* but lacking *phzC* was identified in the SNTKU16 genome, suggesting its potential to produce phenazines. The identification of genes associated with the production of HCN, phenazine, and chitinase in the SNTKU16 genome, along with the positive results from the *in vitro* chitinase, protease and lipase activity assays, suggested a potential for antimicrobial activity and biocontrol applications.

IAA is the principal natural auxin crucial for various aspects of plant development and growth. L–tryptophan is generally considered as the precursor in microbial IAA biosynthesis [44]. In the genome of SNTKU16, genes related to tryptophan biosynthesis (*trpABCDE*) and IAA biosynthesis genes involved in the indole-3-pyruvate pathway (*aldB* and *aspC*) were identified. *In vitro* analysis of IAA production revealed that SNTKU16 produced 28.82 μg/ml of IAA. Furthermore, rice seeds treated with cell suspension and cell-free cultures of SNTKU16 showed significant increases in germination rate, root length, shoot length, and vigor index compared to untreated control seeds. These results indicated that SNTKU16 can promote rice seedling growth, which may be associated with its production of IAA. Similar results have been observed in IAA-producing strains, such as *Pseudomonas* sp. En3 and *P. aeruginosa* B18, which contained the *trpABCDEG* operon related to IAA biosynthesis and have been shown to enhance the growth of *Populus tomentosa* and sugarcane, respectively [6, 45].

Phosphorus is an essential macronutrient for plant growth and is crucial for nutrient cycling in soil systems. However, most soil phosphorus is unavailable to plants because it has low solubility [45]. Phosphate-solubilizing bacteria can convert insoluble soil phosphorus into soluble forms that are accessible to plants. The primary mechanism for phosphate solubilization in pseudomonads is the secretion of gluconic acid, which is derived from glucose through the activity of glucose dehydrogenase and its cofactor, pyrroloquinoline quinone (PQQ) [46]. The SNTKU16 genome contained genes encoding glucose dehydrogenase (*gcd*), genes involved in PQQ cofactor biosynthesis (*pqqC*), and genes for inorganic phosphate solubilization (*ppa*) and organic phosphate mineralization (*ppx*). The enzyme inorganic pyrophosphatase catalyzes the hydrolysis of inorganic pyrophosphate to inorganic phosphate (Pi) [47], while exopolyphosphatase breaks down inorganic polyphosphates into Pi [48]. In bacteria, Pi is transported via two systems: the phosphate inorganic transport (Pit) system and the phosphate-specific transport (Pst) system. Typically, the Pst system includes the proteins PstS, PstC, PstA, and PstB [49], which interact with PhoBR, as well as the phosphate modulator PhoU [50]. The *phoBR* operon is a regulatory system within the Pho regulon that responds to low phosphate concentrations [51] and PhoU plays a crucial role in regulating intracellular phosphate metabolism [52]. Genomic analysis of the SNTKU16 genome revealed genes related to phosphate uptake and transport (*pit* and *pstSCAB*) and genes involved in regulating phosphate starvation responses (*phoBR* and *phoU*). *In vitro* study of SNTKU16 demonstrated its ability to dissolve tri-calcium phosphate in NBRIP medium. This result along with the genome analysis suggested that this strain could offer potential applications in agriculture and biotechnology.

Siderophores are prominent and well-characterized metallophores. In *P. aeruginosa*, pyoverdine and pyochelin are two known siderophores that function as iron-chelating agents by scavenging iron from the extracellular environment [53]. Siderophores not only enhance iron acquisition but also suppress plant pathogen growth through iron competition [54]. The genes involved in pyoverdine synthesis are organized into several distinct clusters. Key genes, including *pvdL*, *pvdI*, *pvdJ*, and *pvdD*, encode non-ribosomal peptide synthetases that are crucial for assembling the pyoverdine molecule. A *pvdE* gene encodes an ATP-binding cassette (ABC) transporter, facilitating the export of pyoverdine. The genes *pvdN*, *pvdO*, *pvdP*, and *pvdQ* play roles in the biochemical maturation processes that lead to chromophore formation [55]. Pyochelin biosynthesis in *P. aeruginosa* involves a complex network of proteins encoded by two divergent operons, *pchDCBA* and *pchEFGHI*. Additionally, the pyochelin-Fe transport operon *fptABCX* is involved in this process [55, 56]. SNTKU16 exhibited siderophore production, with the genome analysis identifying genes involved in synthesizing pyoverdine, such as non-ribosomal peptide synthetases (*pvdL*, *pvdI*, *pvdJ*, and *pvdD*), transporter (*pvdE*), chromophore formation (*pvdN*, *pvdO*, *pvdP*, and *pvdQ*), pyochelin biosynthesis (*pchDCBA* and *pchEFG*) and pyochelin-Fe transport (*fptABCX*). The current study showed that SNTKU16 contained genes involved in synthesizing two siderophores, which are crucial for iron acquisition in microbial systems. This capability likely provides SNTKU16 with a competitive advantage in diverse environments, enhancing its survival and competitiveness in its ecological niche.

Nitrogen is essential for plant growth, but plants cannot directly assimilate atmospheric nitrogen. Instead, nitrogen-fixing bacteria convert atmospheric nitrogen into ammonia, which plants can utilize. This process relies on nitrogen fixation (*nif*) genes, which are required for the maturation of nitrogenase in these bacteria. The *nifU* and *nifS* genes are necessary for the activation of both the Fe protein and MoFe protein in the nitrogenase complex [57]. The genome of SNTKU16 lacked the standard *nif* genes but included non-specific nitrogen fixation genes from the iron-sulfur cluster (ISC) system, suggesting an alternative nitrogen metabolism mechanism. The ISC system encodes the proteins IscU and IscS, which function analogous to NifU and NifS, respectively. In addition, this system features other proteins involved in the maturation of Fe-S proteins, including the alternative scaffold IscA, molecular chaperones HscB and HscA, ferredoxin (Fdx), and the negative regulator IscR [57, 58]. Recently, Deng *et al*. [45] reported the atmospheric nitrogen fixation ability of *Pseudomonas* sp. En3, which contained the *iscU* and *iscS* genes. The presence of genes in the ISC system (*iscU*, *iscS*, *iscA*, *iscR*, *hscB*, *hscA*, *fdx*, and *iscX*) in the SNTKU16 genome suggested that this bacterium may be capable of nitrogen fixation. Additionally, the genome of SNTKU16 included multiple genes involved in nitrogen metabolism, such as *gltBD*, which are involved in ammonia assimilation [59], and *nirBD*, which encodes nitrite reductase associated with nitrite reduction in nitrogen cycling [45]. Ammonia produced by plant growth-promoting microorganisms supplies nitrogen to host plants, leading to increased plant biomass. In the current study, the strain SNTKU16 exhibited positive ammonia production when grown in peptone water broth. This result, along with genome analysis, suggested that SNTKU16 has the potential to enhance plant growth by improving nitrogen availability.

## Conclusion

This study demonstrated that *P. aeruginosa* SNTKU16, isolated from sugarcane field soil, and its cell-free culture had a broad inhibitory effect against the fungal pathogens causing rice dirty panicle and sheath blight diseases. Additionally, the cell suspension and cell-free culture of SNTKU16 showed plant growth promotion activities *in vitro*. These findings should provide a promising foundation for further exploration of the biocontrol and growth-promoting abilities of SNTKU16 under field conditions and its potential application in sustainable agriculture. Genome analysis revealed that this strain has the genetic potential to control plant diseases through both direct and indirect mechanisms. The genome of SNTKU16 will contribute to elucidating the genetic mechanisms underlying its antifungal properties and to developing enhanced biocontrol strategies for managing diseases and promoting plant growth in rice.

## Figures and Tables

**Table 1:** Production of hydrolytic enzymes and plant growth-promoting traits of SNTKU16.

Characteristic	Activity
Amylase production	-
Chitinase production	+
Cellulase production	-
Protease production	+
Lipase production	+
Ammonia production	+
Phosphate solubilization	+ (0.58 ± 0.03 mg/l)
Siderophore production	+ (37.70 ± 2.4 psu)
IAA production	+ (28.82 ± 0.5 μg/ml)

- indicates no activity, + indicates activity

**Table 2:** *In vitro* seed germination, shoot and root lengths, and seed vigor index using cell suspension and cellfree culture of SNTKU16.

Treatment	Germination rate (%)	Shoot length (cm)	Root length (cm)	Seed vigor index (%)
50% Cell suspension	98.90 ± 1.45^a^	4.27 ± 0.40^a^	6.79 ± 0.33^a^	1,093.92 ± 34.32^a^
50% Cell-free culture	98.22 ± 1.62^a^	3.95 ± 0.20^b^	5.33 ± 0.19^b^	911.42 ± 25.50^b^
NB medium	97.54 ± 1.28^b^	2.92 ± 0.23^c^	4.02 ± 0.12^c^	676.98 ± 22.78^c^
Water	96.66 ± 1.83^c^	2.88 ± 0.30^c^	3.10 ± 0.26^d^	578.10 ± 24.79^d^

Values represent mean ± standard deviation. Means within each column followed by different lowercase superscript letters indicate significant differences among treatments at *p* < 0.05.

**Table 3:** General features of SNTKU16 genome.

Feature	Value
Genome size (bp)	6,443,900
GC content (%)	66.3
Genes	5,966
Protein coding sequences (CDSs)	5,899
rRNA genes	3
tRNA genes	61
tmRNA genes	1
Repeat regions	2
CDSs assigned to COGs	5,321

**Table 4:** Genomic relatedness using ANI and dDDH of SNTKU16 with related type species of genus *Pseudomonas*.

Reference genome	ANIb (%)	dDDH (%)	Model CI (%)	Distance
*Pseudomonas aeruginosa* DSM22644^T^	98.69	90.60	88.3–92.4	0.0115
*Pseudomonas aeruginosa* DSM50071^T^	98.60	90.40	88.2–92.3	0.0116
*Pseudomonas aeruginosa* ATCC27853^T^	98.38	89.40	87.0–91.4	0.0128
Pseudomonas paraeruginosa NCTC13628^T^	92.70	52.50	49.8–55.1	0.0661
Pseudomonas tohonis NCTC14580^T^	78.26	23.00	20.7–25.4	0.1906

ANIb values estimated using JspeciesWS software; dDDH value based on formula 2 calculated using the GGDC web server; Model CI, model confidence interval.

**Table 5:** Genes related to biocontrol and plant growth-promoting traits in SNTKU16 genome.